# Assessing Impulsivity in Humans and Rodents: Taking the Translational Road

**DOI:** 10.3389/fnbeh.2021.647922

**Published:** 2021-05-06

**Authors:** Madalena Esteves, Pedro Silva Moreira, Nuno Sousa, Hugo Leite-Almeida

**Affiliations:** ^1^Life and Health Sciences Research Institute (ICVS), School of Medicine, University of Minho, Braga, Portugal; ^2^ICVS/3B’s – PT Government Associate Laboratory, Braga/Guimarães, Portugal; ^3^Clinical Academic Center – Braga, Braga, Portugal; ^4^Psychological Neuroscience Lab, CIPsi, School of Psychology, University of Minho, Braga, Portugal

**Keywords:** impulsivity, behavior, self-report, translation, back-translation

## Abstract

Impulsivity is a multidimensional construct encompassing domains of behavioral inhibition as well as of decision making. It is often adaptive and associated with fast responses, being in that sense physiological. However, abnormal manifestations of impulsive behavior can be observed in contexts of drug abuse and attention-deficit/hyperactivity disorder (ADHD), among others. A number of tools have therefore been devised to assess the different facets of impulsivity in both normal and pathological contexts. In this narrative review, we systematize behavioral and self-reported measures of impulsivity and critically discuss their constructs and limitations, establishing a parallel between assessments in humans and rodents. The first rely on paradigms that are typically designed to assess a specific dimension of impulsivity, within either impulsive action (inability to suppress a prepotent action) or impulsive choice, which implies a decision that weighs the costs and benefits of the options. On the other hand, self-reported measures are performed through questionnaires, allowing assessment of impulsivity dimensions that would be difficult to mimic in an experimental setting (e.g., positive/negative urgency and lack of premeditation) and which are therefore difficult (if not impossible) to measure in rodents.

## Introduction

Impulsivity has been defined in multiple and partially overlapping manners: (i) a tendency to act quickly, although often prematurely, and without appropriate foresight (Dalley and Robbins, 2017), (ii) predisposition to react in a rapid and unplanned manner to internal or external stimuli with reduced consideration for the negative impacts of such reaction (Fineberg et al., 2010), or (iii) a non-reflective stimulus, in opposition to a reward-driven action (Nigg, 2017). It is considered to be in the same spectrum as compulsivity, which can be defined as the repetition of choices or actions in an inflexible manner, despite changes of setting or negative consequences (Voon and Dalley, 2016). However, they are on opposing extremes of a continuum, being assessed with different tests (Hook et al., 2021) and characterized by dissociable psychological and neurological correlates (Voon and Dalley, 2016).

Impulsivity is commonly aggregated into two major categories: impulsive action and impulsive choice. Impulsive action, or rather its inhibition, is the transient suppression of a quick response to an internal or external cue, allowing slower cognitive processes to be able to operate to guide the behavior (Winstanley et al., 2006). It has been suggested that impulsive actions can be divided into two types: action restraint or action cancelation, depending on the action being inhibited before or after its initiation, respectively (Schachar et al., 2007; Eagle et al., 2008). Impulsive choice, on the other hand, implies a decision-making component (Winstanley et al., 2006), mainly in two modalities: temporal discounting and reflection impulsivity. In the first, the preference for immediate smaller over delayed larger rewards reflects a higher impulsive choice. The second is the tendency to make fast decisions in the absence of sufficient evidence (Dalley and Robbins, 2017).

Impulsivity has a major adaptive role, but the balance between impulsivity and inhibition is labile, often depending on the situation. If an object falls off a table, the fast impulsive response of grabbing it is typically beneficial. However, if that object is at an extremely high temperature, such reflex can induce lesion. Such is also true for fast aggressive responses (in war vs. stable society contexts) or choice for a smaller immediate reward over a larger delayed one (in immediate need vs. comfortable living contexts). On the other hand, excessive impulsivity in a given context can lead to negative consequences such as physical injury, problems in maintaining relationships, or even imprisonment. At the pathological level, the *Diagnostic and Statistical Manual of Mental Disorders* (DSM-5) classifies this trait as a diagnosis criterion, a feature, or a risk factor in multiple disorders, including attention-deficit/hyperactivity disorder (ADHD), gambling disorder, and disorders of alcohol or drug use, respectively (American Psychiatry Association, 2013). Indeed, a vast array of literature has associated impulsivity with disorders such as addiction (Mitchell, 2004; Jentsch et al., 2014; Herman and Duka, 2019), reactive aggression (Blair, 2016; Brennan and Baskin-Sommers, 2019), self-harm (McHugh et al., 2019), binge eating disorder (Giel et al., 2017), or ADHD (O’Neill et al., 2017). Comorbidities are also frequent, with impulsivity being a common factor between schizophrenia and aggression (Hoptman, 2015), ADHD and obesity (Cortese et al., 2016), or drug abuse, eating disorders, and self-harm in adolescents (Greydanus and Shek, 2009).

Thus, the assessment of impulsivity in a translational manner is of high importance. In this narrative review, we will systematize behavioral and self-reported measures of impulsivity in a critical manner. Considering that rodents are among the most widely used animal model, we will provide an analysis of tests commonly used for the assessment of impulsivity in humans and rodents and analyze their interspecies comparability. Regarding clinical validity, we will briefly mention results attained in pathologies of altered impulsivity, prioritizing literature with a higher degree of evidence (i.e., meta-analyses and systematic reviews).

## Behavioral Measures of Impulsivity

Behavioral measures have the advantage of evaluating a given dimension of impulsivity in a direct and controlled manner. Also, tests developed for human usage can commonly be adapted for application in laboratory animals and *vice versa*. On the other hand, they are normally unable to assess impulsive behavior that occurs on more complex contexts, which is dependent on a specific emotional or physical state (e.g., impulsivity associated with states of high arousal), thus not capturing all of its dimensions (see self-reported measures for more information).

### Impulsive Action

**FIGURE 1:**
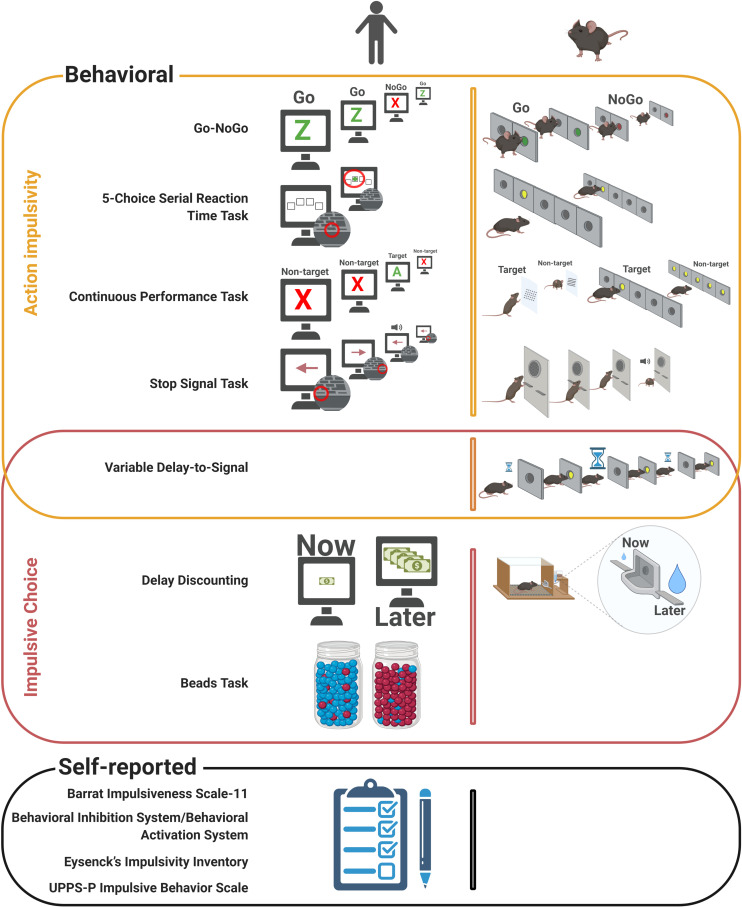
Behavioral and self-reported measures of impulsivity. Behavioral (top) and self-reported (bottom) measures of impulsivity are shown in a schematic manner, highlighting the parallel between human (left) and rodent (right) methods. Image created with BioRender (www.biorender.com).

Tests for assessment of impulsive action typically involve a motor response, whose inhibition is rewarded. Considering their simplicity, they are easily adapted and applied to both rodents and humans (see Table 1 and Figure 1 for direct associations).

**TABLE 1:** Behavioral measures of impulsivity.

**Impulsivity dimension**	**Impulsivity subdimension**	**Human version**	**Rodent version**	**Main construct**
Impulsive action	Action restraint	Go–noGo	Go–noGo	Performing fast responses to Go signals. Withholding the response to a rare noGo signal
		5-csrtt adaptation	5-csrtt	Performing fast and specific responses to stimuli that can be shown at different locations. Avoiding to respond prematurely
		CPT	rCPT and 5C-CPT	Performing fast responses to rare target signals. Withholding the response to a common non-target signal
	Action cancelation	SST	SST	Performing fast responses to a signal. Inhibiting the initiated response upon presentation of a STOP signal
Impulsive choice	Temporal discounting	DD	DD	Choosing between a small immediate reward and a larger, delayed reward
	Reflection impulsivity	Beads task	N/A	Deciding on the amount of information that is sufficient to make a decision
Mixed		N/A	VDS	Performing fast responses to a stimulus. Avoiding to respond prematurely during a stable (impulsive action) or increasing delay (delay intolerance)

*5-csrtt, five-choice serial reaction time task; CPT, continuous performance task; rCPT, rodent continuous performance task; 5C-CPT, five-choice continuous performance task; SST, stop signal task; DD, delay discounting; VDS, variable delay to signal.*

#### Go–noGo Task

The Go–noGo task assesses action restraint. In the *human* version, a sequence of images (typically letters) is shown to the experimental subjects, who are required to press a key whenever the signal is “Go” (e.g., the letter Z). In a subset of the trials, the “noGo” stimulus (instead of the “Go”) is shown, and the subject is required to withhold the response (Winstanley et al., 2006; Winstanley, 2011). Failure to achieve this inhibition is counted as an impulsive response. Many variations of this task have been used, mainly for convenience purposes, or in order to fulfill concerns of each particular study, including using different stimuli (e.g., shapes, pictures, and sounds) (Kaladjian et al., 2011; Dambacher et al., 2015; Hege et al., 2015) or different numbers of Go and noGo stimuli (e.g., V as noGo and any other letter as Go) (Horn et al., 2003). It is also possible to manipulate the number of impulsive responses by altering the presentation proportions (i.e., typically noGo is presented in around 25% of the trials, but this value has been increased up to 50%) (Kaladjian et al., 2011) or by introducing pre-stimulus cues (Kaladjian et al., 2011; Fillmore and Weafer, 2013). Additional elements can be assessed in this test by including distracting images (Brown et al., 2015) or working memory components (Garavan et al., 1999). Because of its simplicity, Go–noGo can be applied to different ages and cognitive profiles. It also seems to be relevant for impulsivity-related disorders, as users of cocaine, MDMA, tobacco, and alcohol were shown to present higher impulsivity in this task, in a meta-analysis (Smith et al., 2014); in contrast, no effects were found in cannabis users, and internet-addicted subjects showed even better inhibitory control than controls (Smith et al., 2014). Also, it has been argued that this test assesses mostly attentional processes (Criaud and Boulinguez, 2013).

Its *rodent* version (also named Go–noGo) follows a similar principle, except that the stimuli are typically sounds or smells. The animal learns that responding to a Go stimulus is reinforced, while responses to the noGo stimulus are not, being considered impulsive. There are multiple variations of this protocol, including different rewards (e.g., food, sucrose, and drugs), cues (e.g., sounds, lights, and smells), apparatus (operant box vs. box with subdivisions), proportion of Go and noGo trials, or application of negative reinforcement (e.g., air puff or quinine). These are typically developed for either convenience purposes (e.g., type of apparatus available) or due to specific experimental concerns (e.g., assessment of the effects of drug or punishment administration on the behavior). Also, protocols in which the animals are head-fixed have been developed, allowing usage of the Go–noGo task in experiments that involve imaging, electrophysiology, or similar protocols (Anker et al., 2009, rats; Moschak and Mitchell, 2012, rats; Jones et al., 2017, rats and mice; Kamigaki and Dan, 2017, mice; Schiff et al., 2018, mice; and Han et al., 2019, mice). In opposition to the human protocols, application in rodents requires extensive training, whose length will depend on the species (mouse or rat) and particularities of the Go–noGo version used. Indeed, these particularities may also need to be adapted according to species. For instance, Jones et al. (2017) showed that rats are able to learn a task in which a positive valence is associated with the Go cue and a negative valence is associated with the noGo cue, but not the opposite, while mice’s learning is associated with the reverse. As in the human version, this task relies on additional functions beyond impulsivity, including attention, Pavlovian and instrumental conditioning, and working memory (Mitchell, 2004).

#### Five-Choice Serial Reaction Time Task

The five-choice serial reaction time task (5-csrtt) is another widely used task for assessment of action restraint in *rodents*. In this task, five response orifices are available, and lights are shown in each one individually. Nose poking in the illuminated hole is rewarded with a sugar pellet, while responses that occur before one of these five lights is on (i.e., during the inter-trial interval [ITI]) are considered impulsive (Carli et al., 1983, rats; Winstanley et al., 2006, rats; Bari et al., 2008, rats; Cope et al., 2016, rats and mice; Higgins and Silenieks, 2017, rats and mice). Also, continuous responses at the apertures after reward delivery are considered perseverative, which are more akin to compulsion, rather than impulsivity (Robbins, 2002). Several variations of this task are used, including alterations in stimulus or ITI duration (altering the propensity toward impulsive responses) or stimulus intensity (altering the attentional demand) (Higgins and Silenieks, 2017, rats). 5-csrtt usage is, however, quite homogenous. Performance on this task is very reliable after appropriate training, which is relatively simple (Higgins and Silenieks, 2017, rats), although prolonged, which may be an impediment for evaluating transient stages of development (e.g., adolescence). Also, other functions are necessarily involved in its performance (e.g., motor, attention, and motivation). Indeed, considering the small stimulus presentation times—down to 1 s or less (Zhong et al., 2018, rats; Bruinsma et al., 2019, rats)—and that the 5-csrtt is based on a human attentional task [continuous performance task (<snm>CPT</snm>); see below] (Winstanley et al., 2006, rats; Higgins and Silenieks, 2017, rats), attentional demand is very high, which can be seen as a potential confounding factor in the assessment of impulsivity, or as an outcome in itself—i.e., the task can be used for attention evaluation (Bari et al., 2008, rats). Of note, rats have been reported to perform more impulsive responses and to be more reliant on temporal than visual strategies (i.e., assess the time to response, instead of relying on aperture lights) compared to mice (Cope et al., 2016).

Regarding a *human* equivalent, despite being based on the CPT, the 5-csrtt is more akin to a recent back-translation to humans as it does not include a key part of the original task—non-target stimuli. Voon et al. (2014) created a direct adaptation of the 5-csrtt, in which four windows in a touch screen are shown. The trial initiates with the subject pressing a computer key, and upon fast presentation of a stimulus in one of the windows, the key must be released and the correct window selected. Premature release of the trial initiation key is considered an impulsive response (Voon et al., 2014). This task was shown, in the same work, to be relevant for subjects with substance dependence (alcohol, tobacco, methamphetamine, and cannabinoids), who presented higher impulsivity levels than controls (Voon et al., 2014). Of notice, an adaptation of this human version has also been developed for application during magnetic resonance imaging (MRI) (Neufang et al., 2016).

#### CPT

The *human* CPT (Rosvold et al., 1956) is very similar to a human Go–noGo with inverted frequencies of “Go” and “noGo” targets. Thus, stimuli are shown sequentially, and upon appearance of a rare target, the subject is required to respond. The main metrics are the error rate and the reaction time (Ballard, 1996; Roebuck et al., 2016). Although some aspects of impulsivity can be captured in this test, it was designed primarily for assessment of attention (Riccio et al., 2002; Roebuck et al., 2016), and that remains its most common usage (Riccio et al., 2002). It is also influenced by other factors that are particularly important in an attentional task and which can be associated with the surrounding environment (e.g., noise and temperature) or with subject state or trait characteristics (e.g., motivation, age, and personality) (Corkum and Siegel, 1993; Ballard, 1996). Multiple variations of this task have been used, including different stimuli (visual letters, auditory letters, or tones), which do not seem to influence the outcome (Roebuck et al., 2016); different targets (e.g., an “X” that is shown after an “A,” thus introducing a working memory component); frequencies of target presentation; or interstimulus intervals (altering the attentional component and the propensity for impulsive responses) (Riccio et al., 2002). In meta-analyses, the CPT showed good performance in the assessment of children with ADHD (Losier et al., 1996; Huang-Pollock et al., 2012), as well as in the distinction between treated and non-treated patients with this pathology (Losier et al., 1996).

The CPT has been adapted for *rodent* usage in a multitude of manners. The most commonly used for impulsivity assessment is the 5-csrtt (see section “5-Choice Serial Reaction Time Task”), although the rodent CPT (rCPT) and the 5C-CPT are more akin to the original task. As in the CPT, rodents performing the rCPT are required to respond to target stimuli and to withhold response to non-targets. The task is administered using a touch screen, where a pattern is shown. The animal should touch a target pattern and withhold response to a non-target pattern in order to receive a reward; each stimulus is presented 50% of the time (Kim et al., 2015, mice). Of note, as an adaptation of the CPT, the rCPT was developed as a task to assess attention (Kim et al., 2015, mice) rather than impulsivity, although it has also been used for that effect (Caballero-Puntiverio et al., 2019, mice). The five-choice CPT (5C-CPT; rodents) is very similar to the above-described 5-csrtt, with the target being a light that is shown in one of five apertures, but also including a non-target stimulus—the simultaneous presentation of all five lights—to which the animal must withhold response (Bhakta and Young, 2017, rats and mice; Higgins and Silenieks, 2017, rats). It has been developed aiming to assess attention, vigilance, and response inhibition (Bhakta and Young, 2017), although it is not commonly used, potentially due to being so recent (2017).

#### Stop Signal Task

In opposition to the above-mentioned tests which are designed to evaluate action restraint, the stop signal task (SST) is the only one able to assess action cancelation. In its typical format for *human* application, a sequence of visual stimuli is shown, to which one of two actions is requested (e.g., pressing a left button for a leftward arrow and a right button for a rightward arrow). In some trials (typically 25%) and at random delays, a second stimulus (e.g., an image of a cross or a tone) is presented after the first, signaling that the response must be inhibited (stop signal) (Smith et al., 2014; Verbruggen et al., 2019). The stop-signal reaction time is estimated from the probability of stopping upon presentation at different delays and reflects the time that is required to stop an initiated response (Smith et al., 2014). Multiple adaptations of the SST have been used, aiming to alter either the attentional load or the stop reaction time. These include alterations of the number of stimuli/responses in the “Go” condition, salience of the stop signal, or frequency of presentation of the stop signal. Aiming to homogenize conclusions, a consensus guide was recently published (Verbruggen et al., 2019). The construct of this task allows reduced interference of attention in the assessment of impulsivity, and several meta-analyses have found effects in pathologies typically associated with impulsivity, including ADHD (Alderson et al., 2007; Lipszyc and Schachar, 2010), pathological gambling (Smith et al., 2014; Chowdhury et al., 2017) (although Lipszyc and Schachar, 2010, did not find this effect), substance dependence (Lipszyc and Schachar, 2010), and schizophrenia (Lipszyc and Schachar, 2010). On the other hand, as SST is designed to elicit approximately 50% failures, it may be considered too difficult by some subjects and decrease motivation (Smith et al., 2014).

The *rodent* version of SST follows a similar construct. The animals are trained to, after a signal, press a first and then a second lever, sequentially, in order to receive a reward. On some trials (typically 20%), a tone (stop signal) is presented after pressing of the first lever, and the animal is required to withhold from pressing the second lever (Winstanley and Clark, 2016, rats). Because the task is very similar to the human one, impulsive-like behavior can easily be inferred; however, it requires extensive training.

### Impulsive Choice

Tests for assessment of impulsive choice imply a decision between two potential actions, aiming to maximize the attained reward. Such implies complex processes which often hinder a direct translation of tasks between human and rodents (see Table 1 and Figure 1 for direct associations).

#### Delay Discounting

The delay discounting (DD) task is based on the assessment of reward value through a balance between its size and the delay to get it. Typically, across trials, one of these two variables is changed, eventually reaching a level at which the value of both rewards is similar (indifference point) (Vanderveldt et al., 2016). In *humans*, the task is often performed using a computerized platform. Variations may depend on the goal of the study, e.g., different types of reward (money, drugs, food, etc.), or aim to alter the levels of impulsive decision making by changing the sequence in which the values are presented (ascending, descending, balanced, or random—see Robles and Vargas, 2008, for effects) or the number/size of delays/rewards (da Matta et al., 2012). Also, the structure can be fixed for all participants or adapted to the performance, aiming to increase sensitivity (da Matta et al., 2012). Steeper discounting has been associated with obesity (Amlung et al., 2016) and abuse of alcohol, tobacco, cannabis, stimulants, opiates, or gambling (Amlung et al., 2017). While testing is simple and data analysis is well established, its simplicity in comparison with real-world decision making has been discussed (Vanderveldt et al., 2016). Indeed, the task typically contemplates one immediate reward and one associated with a delay, but not two different delays. Such would be associated with more complex processes of decision, in which a preference reversal is commonly observed, i.e., an initial preference for the larger, more delayed reward, which is reversed as the time of the smallest, less delayed one gets closer (e.g., the planning for starting a diet on Monday, aiming to achieve a healthier lifestyle, which is replaced by the desire for highly energetic food) (Vanderveldt et al., 2016).

In the *rodent* version, the animals are required to select one of two levers in order to receive the corresponding reward (typically sugared food) (Winstanley et al., 2006, rats). In alternative, it has been performed in a T-maze, instead of an operant box (i.e., the choice is made by selecting the left or the right arm, rather than a lever or a nose-poke hole) (Winstanley, 2011, rats; Feja et al., 2014, rats; Masuda et al., 2020, mice), but other variations include the way the delay evolves (preset or adjusting and within or between sessions), the type of reward (e.g., food or drugs), or its relative size (Winstanley, 2011, rats).

While the human and rodent tasks are quite similar in their construction, they often differ in key aspects, including (i) the type of reward—palatable food or drink, alcohol, or drugs in rodents—and hypothetical money (most common), real food/drink, real money, entertainment, activities, or social/sexual reward in humans (of note, non-monetary rewards used in humans have been shown to increase non-systematic responding, Smith et al., 2018); (ii) the delay—normally in the range of seconds for rodents and months or years in humans (although delays of seconds have been previously applied to humans, Pietras et al., 2003); (iii) the reward presentation—typically, animals experience the reward, while it is just communicated to humans; (iv) the waiting—rodents who choose the long delay have to endure it in a relatively small space with minimal entertainment, while humans are able to proceed with their normal activities during the delay (Vanderveldt et al., 2016).

#### Beads Task

The beads task, used in *humans*, assesses the “jumping to conclusions” bias, which is considered as a lack of reflection impulsivity, even though it is controversial whether this is a test of impulsive choice. In this task, two jars of beads are filled with equal but opposite ratios of different-colored beads (e.g., jar 1 has 85 red and 15 blue beads, and jar 2 has 85 blue and 15 red beads). The jars are hidden, and individual beads are shown in a predetermined order to the subject, who needs to decide from which jar the beads are being taken. Two main variables are assessed: the number of beads drawn before a decision is made, and the proportion of extreme responders (i.e., subjects who make a decision based on one or two beads) (Dudley et al., 2016). Common variations of the beads task include changes in the ratios or in the jar contents (Dudley et al., 2016), altering impulsive decision; or inclusion of distractor sequences (McLean et al., 2018), improving reliability and repeatability (McLean et al., 2018). In a meta-analysis, people with psychosis in comparison with healthy controls required less beads to make a decision and had a higher number of extreme responders. Also, people with delusions required less beads than people with psychosis but without delusions, also having more extreme responders (Dudley et al., 2016). In impulsivity-related pathologies, to the best of our knowledge, no systematic reviews or meta-analyses were performed, although data suggest alterations in this task in binge drinkers (Banca et al., 2016). There is no rodent equivalent of this task, nor is there a rodent task that claims to assess reflection impulsivity.

### Mixed Tasks

One *rodent* task, the variable delay to signal (VDS), assesses both impulsive action and delay tolerance (impulsive choice) (Leite-Almeida et al., 2013, rats; Soares et al., 2018, rats). It was originally based on the 5-csrtt, but using only one response aperture. The animals are required to nose-poke in the aperture when its light is on but refrain from doing it in the delay that precedes presentation of such light (impulsive response). At a first stage of the task, this delay is maintained constant (3 s), and premature responses reflect action impulsivity (i.e., were associated with the 5-csrtt). The second stage includes three blocks of different delays: 3, 6/12, and again 3 s, and an increase in impulsive responses in consequence of the larger delays reflects delay intolerance (i.e., is associated with DD) (Leite-Almeida et al., 2013, rats; Soares et al., 2018, rats). To date, no variations of this task have been published, except for an adaptation of the delays after the first publications (Leite-Almeida et al., 2012, rats; Leite-Almeida et al., 2013, rats), which may be due to a utilization restricted to the original group (Leite-Almeida et al., 2012, rats; Leite-Almeida et al., 2013, rats; Melo et al., 2016, rats; Carvalho et al., 2017, rats; Cunha et al., 2017, rats; Soares et al., 2018, rats; Cunha et al., 2020a, rats; Cunha et al., 2020b, rats), with only one exception to date (Jiménez-Urbieta et al., 2020, rats). In comparison with other tasks, the VDS reduces the attentional bias, as well as potential effects of extensive training. Its reduced training time (7 days) also allows assessment of transient states (e.g., adolescence). Indeed, it was shown to be sensitive to age, sex, and strain differences (Soares et al., 2018, rats). On the other hand, it still relies on motor performance, and despite association with DD, its inclusion as a task for the assessment of choice impulsivity would be discussible, as it does not imply a choice *per se*, but rather a delay intolerance component. Although the VDS is based on the 5-csrtt (which is, in turn, based on the CPT), it does not have a direct human equivalent task, nor has it been used in mice.

## Self-Reported Measures of Impulsivity

Self-reported measures of impulsivity are attained using structured questionnaires regarding attitudes or feelings in different situations. Such allows the assessment of impulsivity within given contexts that cannot be reliably reproduced in a laboratory, and despite this dimension of subjectivity, they have often shown reliability and reproducibility (see below). On the other hand, subjective measures cannot be back-translated to rodents. We here summarize some of the most commonly used scales. For a more in-depth analysis of self-reported measures, please consult the recent review by Hook et al. (2021). Table 2 summarizes the basic components of the here-described tests.

**TABLE 2:** Self-reported measures of impulsivity.

**Task**	**Response scale**	**No. of items**	**Impulsivity dimensions**
BIS-11	1 (rarely/never) to 4 (almost always/always)	30	Attentional (attention and cognitive instability), motor (motor and perseverance) and non-planning impulsiveness (self-control and cognitive complexity)
BIS/BAS	1 (very true for me) to 4 (very false for me)	24	BIS and BAS (drive, reward, and fun seeking)
Eysenck’s Impulsivity Inventory	Yes/No	54	Impulsiveness, venturesomeness, and empathy
UPPS-P	1 (agree strongly) to 4 (disagree strongly)	59	Negative urgency, positive urgency, sensation seeking, lack of premeditation, and lack of perseverance

*BIS-11, Barratt Impulsiveness Scale-11; BIS, Behavioral Inhibition System; BAS, Behavioral Activation System; UPPS-P, Impulsive Behavior Scale.*

### Barratt Impulsiveness Scale-11

The Barratt Impulsiveness Scale-11 (BIS-11) is one of the most widely used scales for the assessment of impulsivity. Its currently used version was designed by Patton et al. (1995) and assesses three main factors within 30 items, which can be further subdivided: attentional impulsiveness (attention and cognitive instability), motor impulsiveness (motor and perseverance), and non-planning impulsiveness (self-control and cognitive complexity), to which the subject responds through a scale that ranges from 1 (rarely/never) to 4 (almost always/always). Attentional impulsiveness items include statements such as “I don’t pay attention” or “I am a steady thinker” (inverted). Motor impulsiveness is reflected in sentences such as “I act on impulse” or “I am future oriented” (inverted). Non-planning impulsiveness is assessed though statements such as “I say things without thinking” or “I like to think about complex problems” (inverted) (International Society for Research on Impulsivity, 2020). Meta-analyses have shown that <snm>BIS</snm>-11 motor impulsivity is altered in pathological gamblers (Chowdhury et al., 2017) and that all dimensions are altered in bipolar disorder (Saddichha and Schuetz, 2014). Also, a systematic review has shown an association with food addiction (Maxwell et al., 2020).

### Behavioral Inhibition System/Behavioral Activation System Scale

The Behavioral Inhibition System/Behavioral Activation System (BIS/BAS) scale was developed by Carver and White (1994) and is based on the idea of two contrasting systems. One is associated with anxiety, is sensitive to negative outcomes, and is activated do avoid them (BIS), while the second is associated with appetitive motivation, is sensitive to positive outcomes, and is activated to approach them (Carver and White, 1994). This scale includes 24 items to which the subject responds in a scale that ranges from 1 (very true for me) to 4 (very false for me). BIS/BAS includes four subscales: (i) BIS, assesses the reaction to an anticipated punishment through sentences as “I worry about making mistakes” or “Criticism and scolding hurts me quite a bit”; (ii) BAS drive, directed at the pursuit of desired goals, including “I go out of my way to get things that I want”; (iii) BAS reward responsiveness, assesses the positive response to the anticipation of a reward, such as “When I get something I want, I feel excited and energized”; and (iv) BAS fun seeking, evaluates the desire for new rewards and the approach motivation toward potentially rewarding events, such as “I will often do things for no other reason than that they might be fun” or “I often act in the spur of the moment” (Carver and White, 1994). Despite its wide usage, to the best of our knowledge, no meta-analyses have been published to assess BIS/BAS effects on impulsivity-related disorders. One systematic review, however, was unable to find associations of BAS and food addiction, while the number of BIS studies was insufficient for conclusion withdrawal (Maxwell et al., 2020). Nonetheless, the literature suggests associations with alcohol (Studer et al., 2016) and nicotine (Baumann et al., 2014) use.

### Eysenck’s Impulsivity Inventory

Eysenck’s Impulsivity Inventory, also known as the Impulsiveness, Venturesomeness, and Empathy (IVE) Questionnaire, was developed, in its current version (I7) by Eysenck et al. (1985). Although it is not as commonly used as the above-mentioned scales, we include it in this review due to its different construct. The questionnaire is composed of 54 items to which the subjects respond in a dichotomic manner (yes or no). These are organized into three subscales: (i) impulsiveness, including items as “Do you often buy things on impulse?” or “Before making up your mind, do you consider all the advantages and disadvantages?” (inverted); (ii) venturesomeness, including “Would you enjoy water skiing?” or “Do you find it hard to understand people who risk their necks climbing mountains?” (inverted); and (iii) empathy, including “Would you feel sorry for a lonely stranger?” or “Do you like watching people open presents?” (Eysenck et al., 1985). No meta-analyses or systematic reviews have assessed this scale’s results in impulsivity-related disorders, although data suggest an association with obsessive-compulsive (Smári et al., 2008) and borderline personality (Mortensen et al., 2010) disorders, MDMA consumption (Morgan, 1998), and binge eating (Cuzzocrea et al., 2015).

### UPPS-P Impulsive Behavior Scale

More recently, the UPPS-P Impulsive Behavior Scale was developed, having the particularity of assessing impulsive behavior that occurs under extreme positive emotions, i.e., positive urgency (Cyders et al., 2007). It is composed of 59 items (a shorter version of 20 items has also been developed, Cyders et al., 2014), to which answers range from 1 (agree strongly) to 4 (disagree strongly). Five dimensions are assessed: (i) negative urgency, including “When I feel rejected, I will often say things that I later regret” (inverted); (ii) positive urgency, as in “When I am in great mood, I tend to get into situations that could cause me problems” (inverted); (iii) sensation seeking, including “I quite enjoy taking risks” (inverted); (iv) lack of premeditation, such as “I like to stop and think things over before I do them”; and (v) lack of perseverance, including “Unfinished tasks really bother me” (International Society for Research on Impulsivity, 2020). Meta-analyses have been able to find associations between these subscales and impulsivity-related disorders, including alcohol (Berg et al., 2015) and substance (Berg et al., 2015; VanderVeen et al., 2016) abuse, nicotine dependence (Kale et al., 2018; Bos et al., 2019), borderline personality traits, suicidality, aggression, and eating disorders (Berg et al., 2015).

## Discussion

Impulsivity is a multifaceted construct influenced by both biological (e.g., genetic, age, and sex) and environmental (familial, cultural, etc.) factors. For instance, impulsivity has been reported as sex dependent in both humans (Cross et al., 2011; Weafer and de Wit, 2014) and rodents (Weafer and de Wit, 2014; Soares et al., 2018), but the direction and strength of such effect depend on the assessed dimension. Additional variability may arise due to the mediation of other factors, as hormonal cycle in women (Diekhof, 2015) and female rats (Swalve et al., 2016), age (Soares et al., 2018, rats; Rosenbaum and Hartley, 2019, humans), genetics (Bezdjian et al., 2011, humans; Soares et al., 2018, rats; Jupp et al., 2020, rats), or environment (Bezdjian et al., 2011, humans; Kirkpatrick et al., 2013, rats). These influences, as well as their human–rodent parallels, are of high importance for the development of translational research. One additional relevant factor is attention, which is required for all the behavioral tasks presented above. It is widely acknowledged that attention to new environmental cues is critical for inhibiting the current flow of decisions and actions and for shifting toward a more appropriate flow (Bari and Robbins, 2013). Nevertheless, some of the available behavioral tasks for the assessment of impulsivity are frequently criticized by their excessive focus on attentional demand (e.g., 5-csrtt).

The here-described methods cover the spectrum of impulsivity dimensions and have been shown to reliably detect alterations in impulsivity in clinical contexts in which it is expected to be altered, including drugs (Jentsch et al., 2014) or alcohol (Herman and Duka, 2019) abuse, smoking (Mitchell, 2004), reactive aggression (Blair, 2016; Brennan and Baskin-Sommers, 2019) (often in the context of schizophrenia, Hoptman, 2015), self-harm (McHugh et al., 2019), binge eating disorder (Giel et al., 2017), or ADHD (O’Neill et al., 2017)—see text for details on meta-analyses and systematic reviews. Rodent task validation, on the other hand, is partially assumed by the similarities to their human counterparts, as most of these contexts can only be partially replicated in rodents. However, all the here-described rodent tasks have shown alterations of impulsivity associated with substances of abuse (e.g., 5-csrtt, Broos et al., 2017; 5C-CPT, Irimia et al., 2014; SST, Beckwith and Czachowski, 2016; DD, Harvey-Lewis et al., 2014; and VDS, Leite-Almeida et al., 2013).

Two main types of impulsivity assessments are here presented: behavioral and self-reported measures. Even though impulsivity is expected to vary with age, self-reported measures assess impulsivity as a trait—i.e., the scores are expected to remain relatively stable over time. They present several advantages, including the ability to assess different dimensions of impulsivity in the same questionnaire, which is applied in 5–15 min without the need for any equipment. They demonstrate satisfying psychometric characteristics, including good internal consistency and high test–retest reliability, and provide a context for the evaluated behaviors (e.g., “When I am in great mood, I tend to get into situations that could cause me problems” in UPPS-P). However, these behaviors are evaluated according to the subject’s perception and are thus not necessarily objective and of limited application if self-perception is altered. Importantly, self-reported measures are not transposable to animals. On the other hand, behavioral assessments provide laboratory-controlled, objective measurements of a given impulsivity dimension. These measurements can be altered according to internal states (e.g., arousal and stress) and can thus be considered more akin to state impulsivity, being therefore more suitable for association with transient states (e.g., drug effects). They can often be paralleled between humans and rodents, allowing a translational evaluation of cellular, molecular, and network players. However, they fail to provide more complex contexts to the assessment. A concern related to these laboratorial assessments pertains to a limited external validity, particularly in animal models. On the other hand, the use of imagetic and simulated settings (e.g., the use of virtual reality, Pollak et al., 2009; Henry et al., 2012) is a recurrent strategy that diminishes this limitation on the behavioral assessment of impulsivity in human subjects. Of note is that the concordance between self-report and behavioral measures seems to be weak, suggesting that they are assessing different constructs (see for example, Cyders and Coskunpinar, 2012; Hasegawa et al., 2019).

One additional source of variability between studies is the multiple adaptations of established tasks for both humans and rodents. These are often performed in order to answer a specific experimental question (e.g., impulsivity when aiming to attain drugs instead of natural rewards) or to adapt the task to a specific population (e.g., using images instead of letters when studying a cohort of children or manipulating the number of Go and noGo trials in a population with altered attention). Although often necessary, these changes from the originally designed task create difficulties in comparability and may raise questions regarding validity, as validation of small changes is rarely performed. Even though several systematic reviews of the literature focusing on impulsivity exist—see for example (Smith et al., 2014) for Go–noGo and SST—they typically do not account for task variations, which may provide relevant insights regarding the most adequate manipulations to assess impulsivity under specific conditions/goals. Also, behavioral measures have been adapted from humans to rodents and *vice versa*. Such implies alteration in the structure of the task, including the applied stimuli (e.g., letters for humans and sounds for animals) or the rewards (e.g., hypothetical money for humans and physical sugar pellets for rodents). These adaptations are necessary, although interpretations of a parallel between human and rodent behaviors require careful consideration. Indeed, one can argue that the behavioral tasks here presented for rodents try to mimic human impulsivity in species who would not naturally present these behaviors. Reactive aggression, for example, is an ethological behavior in both humans and rodents, and being associated with impulsivity (Blair, 2016; Brennan and Baskin-Sommers, 2019), it could be used to establish this parallel.

There are also additional limitations that are different in rodents and humans. In rodents, whenever the reward is palatable food, caloric restriction is necessary, creating difficulties in the interpretation of impulsivity vs. satiety. Also, in rodents (and to a smaller degree in humans), there is a very high motor demand in some of the tasks, as 5-csrtt, where fast, premature responses are considered impulsive, hindering interpretation or requiring adaptation of the task for aged or injured animals. On the other hand, animal experiments are typically performed by a comparison of different groups (e.g., cortical lesion vs. controls), who should all be in the same satiety conditions and whose motor performance can be assessed and controlled for. In human subjects, however, a range of additional factors can influence impulsivity (see above), some of which, as cultural or familial environment, can be difficult to control.

## Conclusion

Impulsivity can be considered as an umbrella term in which multiple processes are included. Importantly, not all forms of impulsivity can be objectively measured through laboratory-controlled tasks, whose complementarity with self-reported measures is evidenced by the poor correlation between them. Such self-reported measures, as well as some behavioral ones (e.g., assessment of lack of planning), imply complex reasoning that, even if potentially adaptable to rodent behavioral tasks, would be hard to interpret. Indeed, even in simpler tasks, such as the DD, there is a necessary adaptation of times and rewards, which brings out questions regarding the validity of such translation. These difficulties are added to the necessary consideration of additional factors whose disentanglement from the impulsive behavior is not clear, including attention, memory, and motivation, as well as the context in which the impulsive behavior occurs (e.g., urgency in positive or negative situations). There is, however, an evident effort to establish a parallel between tasks, creating multiple translations and back-translations and thus allowing the assessment of the core of the behavior in a translational manner.

## Author Contributions

ME performed the literature research. ME and HL-A designed the manuscript. ME, PM, and HL-A wrote the first draft. ME, PM, NS, and HL-A discussed, provided input, and approved the final manuscript. All authors contributed to the article and approved the submitted version.

## Conflict of Interest

The authors declare that the research was conducted in the absence of any commercial or financial relationships that could be construed as a potential conflict of interest.
